# Crystal structure and Hirshfeld surface analysis of aqua­bis­(nicotinamide-κ*N* ^1^)bis­(2,4,6-tri­methyl­benzoato-κ^2^ *O*,*O*′)cadmium(II)

**DOI:** 10.1107/S2056989018001494

**Published:** 2018-01-31

**Authors:** Tuncer Hökelek, Safiye Özkaya, Hacali Necefoğlu

**Affiliations:** a Department of Physics, Hacettepe University, 06800 Beytepe, Ankara, Turkey; b Department of Chemistry, Kafkas University, 36100 Kars, Turkey; c International Scientific Research Centre, Baku State University, 1148 Baku, Azerbaijan

**Keywords:** crystal structure, cadmium(II), transition metal complexes of benzoic acid and nicotinamide derivatives

## Abstract

The asymmetric unit of the title complex, [Cd(C_10_H_11_O_2_)_2_(C_6_H_6_N_2_O)_2_(H_2_O)], contains one half of the complex mol­ecule, with the Cd^II^ cation and the coordinated water O atom residing on a twofold rotation axis. The Cd^II^ cation is coordinated in a bidentate manner to the carboxyl­ate O atoms of the two symmetry-related 2,4,6-tri­methyl­benzoate (TMB) anions and to the water O atom at distances of 2.297 (2), 2.527 (2) and 2.306 (3) Å to form a distorted penta­gonal arrangement, while the distorted penta­gonal–bipyramidal coordin­ation sphere is completed by the two pyridine N atoms of the two symmetry-related monodentate nicotinamide (NA) ligands at distances of 2.371 (3) Å in the axial positions. In the crystal, mol­ecules are linked *via* inter­molecular N—H⋯O, O—H⋯O and C—H⋯O hydrogen bonds with *R*_2_^2^(12), *R*_3_^3^(8), *R*_3_^3^(14), *R*_3_^3^(16), *R*_3_^3^(20), *R*_3_^3^(22), *R*_4_^4^(22), *R*_5_^5^(16), *R*_6_^6^(16) and *R*_6_^6^(18) ring motifs, forming a three-dimensional architecture. The Hirshfeld surface analysis of the crystal structure indicates that the most important contributions for the crystal packing are H⋯H (56.9%), H⋯C/C⋯H (21.3%) and H⋯O/O⋯H (19.0%) inter­actions.

## Abstract 2

The Cd^II^ cation, located on a twofold rotation axis, is coordinated by two 2,4,6-tri­methyl­benzoate anions, two nicotinamide ligands and a water mol­ecule in a distorted penta­gonal–bipyramidal geometry.

## Chemical context   

Nicotinamide (NA) is one form of niacin. A deficiency of this vitamin leads to loss of copper from the body, known as pellagra disease. Victims of pellagra show unusually high serum and urinary copper levels (Krishnamachari, 1974 ▸). The crystal structure of NA was first determined by Wright & King (1954 ▸). The NA ring is the reactive part of nicotinamide adenine dinucleotide (NAD) and its phosphate (NADP), which are the major electron carriers in many biological oxidation–reduction reactions (You *et al.*, 1978 ▸). The nicotinic acid derivative *N*,*N*-di­ethyl­nicotinamide (DENA) is an important respiratory stimulant (Bigoli *et al.*, 1972 ▸).

Transition metal complexes with ligands of biochemical inter­est such as imidazole and some N-protected amino acids show inter­esting physical and/or chemical properties, through which they may find applications in biological systems (Antolini *et al.*, 1982 ▸). Crystal structures of metal complexes with benzoic acid derivatives have been reported extensively because of the varieties of the coordination modes. For example, Co and Cd complexes with 4-amino­benzoic acid (Chen & Chen, 2002 ▸), Co complexes with benzoic acid (Catterick *et al.*, 1974 ▸), 4-nitro­benzoic acid (Nadzhafov *et al.*, 1981 ▸) and phthalic acid (Adiwidjaja *et al.*, 1978 ▸), and Cu complexes with 4-hydro­chloric acid (Shnulin *et al.*, 1981 ▸) have been described.

The structure–function–coordination relationships of the aryl­carboxyl­ate ion in Cd^II^ complexes of benzoic acid deriv­atives change depending on the nature and position of the substituted groups on the benzene ring, the nature of the additional ligand mol­ecule or solvent, and the pH and temperature of synthesis (Shnulin *et al.*, 1981 ▸). When pyridine and its derivatives are used instead of water mol­ecules, the structure is completely different (Catterick *et al.*, 1974 ▸).

The structures of some mononuclear complexes obtained from the reactions of transition metal(II) ions with nicotinamide (NA) and some benzoic acid derivatives as ligands have been determined previously, *e.g.* [Zn(C_7_H_5_O_3_)_2_(C_6_H_6_N_2_O)_2_] [(II); Necefoğlu *et al.*, 2002 ▸], [Mn(C_7_H_4_ClO_2_)_2_(C_10_H_14_N_2_O)_2_(H_2_O)_2_] [(III); Hökelek *et al.*, 2008 ▸], [Zn(C_8_H_8_NO_2_)_2_(C_6_H_6_N_2_O)_2_]·H_2_O [(IV); Hökelek *et al.*, 2009*a* ▸], [Mn(C_9_H_10_NO_2_)_2_(C_6_H_6_N_2_O)(H_2_O)_2_] [(V); Hökelek *et al.*, 2009*b* ▸] and [Co(C_9_H_10_NO_2_)_2_(C_6_H_6_N_2_O)(H_2_O)_2_] [(VI); Hökelek *et al.*, 2009*c* ▸]. The structure determination of the title compound, (I), a cadmium complex with two 2,4,6-tri­methyl­benzoate (TMB) and two nicotinamide (NA) ligands and one coordinated water mol­ecule, was undertaken in order to compare the results obtained with those reported previously. In this context, we synthesized the Cd^II^-containing title compound and report herein its crystal and mol­ecular structures along with the Hirshfeld surface analysis.
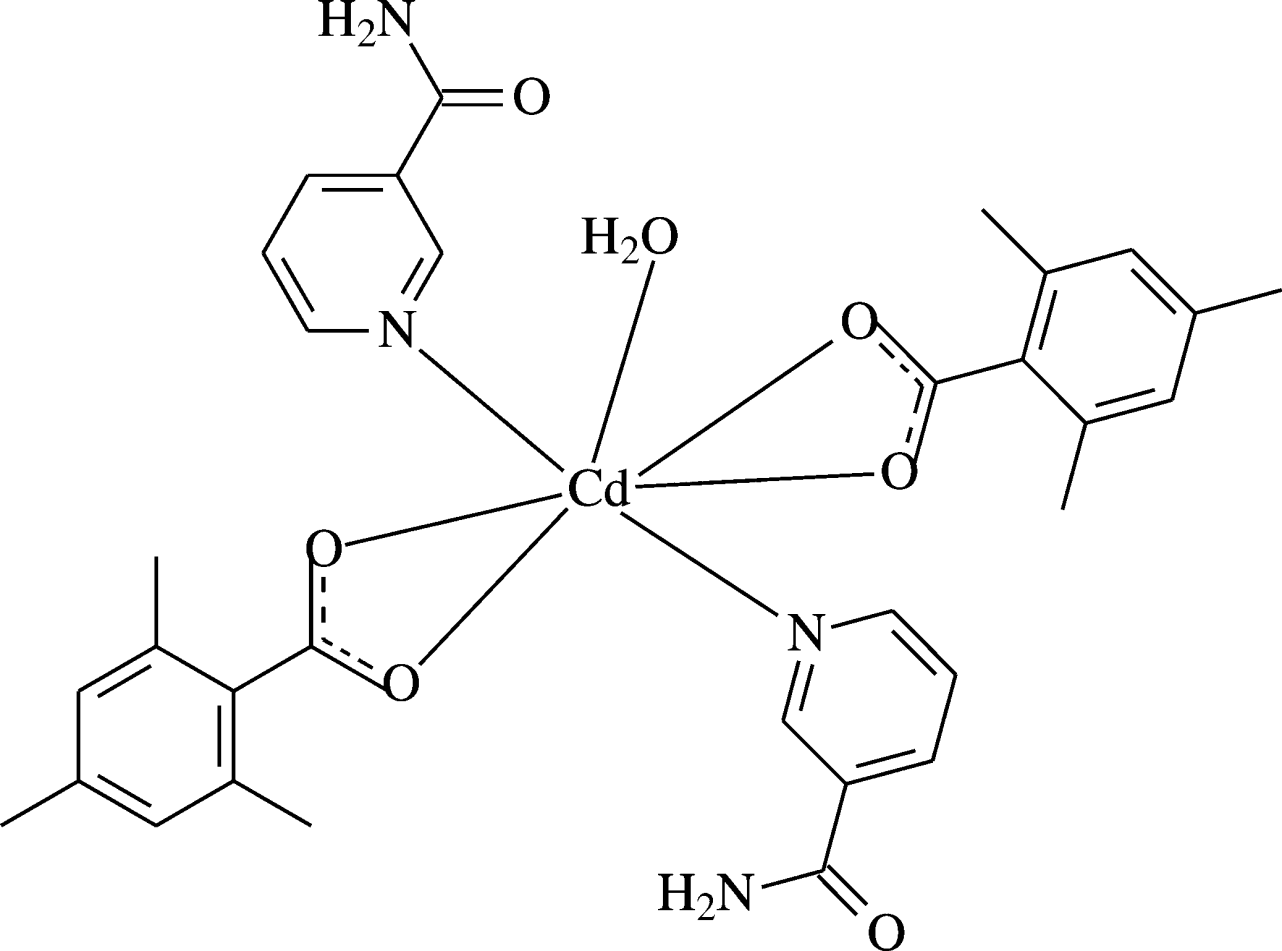


## Structural commentary   

**Figure 1:**
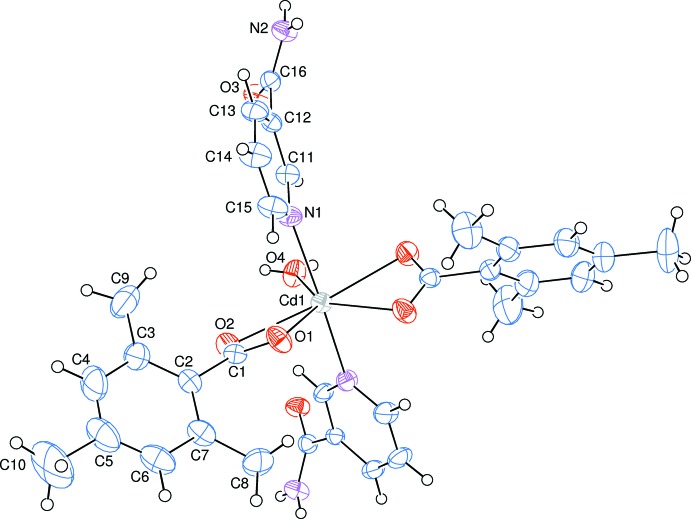
The mol­ecular structure of the title complex with the atom-numbering scheme. Unlabelled atoms are related to labelled atoms by the symmetry operation (1 − *x*, *y*, 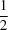 − *z*). Displacement ellipsoids are drawn at the 50% probability level.

The asymmetric unit of the crystal structure of the mononuclear title complex contains half of a Cd^II^ cation (site symmetry 2), one 2,4,6-tri­methyl­benzoate (TMB) anion and one nicotin­amide (NA) mol­ecule together with half of a water mol­ecule (point group symmetry 2), the TMB and NA ligands coord­inating in bidentate and monodentate manners, respectively (Fig. 1 ▸).

The Cd^II^ cation is coordinated bidentately to the carboxyl­ate O atoms (O1, O2, O1^i^ and O2^i^) of two symmetry-related 2,4,6-tri­methyl­benzoate (TMB) anions and to the water O atom (O4) at distances of 2.297 (2), 2.527 (2) and 2.306 (3) Å, respectively, to form a distorted penta­gonal arrangement. The sum of the bond angles O1—Cd1—O1^i^ [87.57 (11)°], O1—Cd1—O2 [53.63 (7)°], O1^i^—Cd1—O2^i^ [53.63 (7)°], O2—Cd1—O4 [84.47 (5)°] and O2^i^—Cd1—O4 [84.47 (5)°] in the basal plane around Cd^II^ cation is 363.77° [symmetry code: (i) 1 − *x*, *y*, 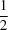 − *z*]. This confirms the presence of the Cd^II^ cation with a small deviation from the basal plane. The distorted penta­gonal–bipyramidal coordination sphere is completed by the two pyridine N atoms (N1 and N1^i^) of the two symmetry-related monodentate nicotinamide (NA) ligands at distances of 2.371 (3) Å in the axial positions (Fig. 1 ▸).

The near equalities of the C1—O1 [1.249 (4) Å] and C1—O2 [1.253 (3) Å] bonds in the carboxyl­ate groups indicate delocalized bonding arrangements, rather than localized single and double bonds. The O2—C1—O1 bond angle [121.7 (3)°] seems to be slightly decreased than that present in a free acid [122.2°]. The O2—C1—O1 bond angle may be compared with the corresponding values of 123.5 (2) and 120.4 (2)° in (II), 125.2 (5)° in (III), 119.2 (3) and 123.8 (2)° in (IV), 123.6 (3) and 119.4 (3)° in (V) and 123.86 (13) and 118.49 (14)° in (VI), where the benzoate ions are coordinated to the metal atoms only monodentately in (III), and both monodentately and bidentately in (II), (IV), (V) and (VI). The Cd1 atom lies 0.0192 (1) Å above of the planar (O1/O2/C1) carboxyl­ate group. The O1—Cd1—O2 angle is 53.63 (7)°. The corresponding O—*M*—O angles are 58.79 (6)° in (II), 59.02 (8)° in (IV), 58.45 (9)° in (V) and 60.70 (4)° in (VI). In the TMB anion, the carboxyl­ate group is twisted away from the attached benzene ring, *A* (C2–C7), ring by 60.94 (18)°, while the benzene and pyridine rings [pyridine = *B* (N1/C11–C15)], are oriented at a dihedral angle of 50.32 (11)°. The four-membered ring *D* (Cd1/O1/O2/C1) is nearly planar with a maximum deviation of 0.0029 (30) Å (for C1) from the mean plane, and it is oriented at dihedral angles of 60.98 (11) and 81.91 (7)°, with respect to the *A* and *B* rings.

## Supra­molecular features   

**Figure 2:**
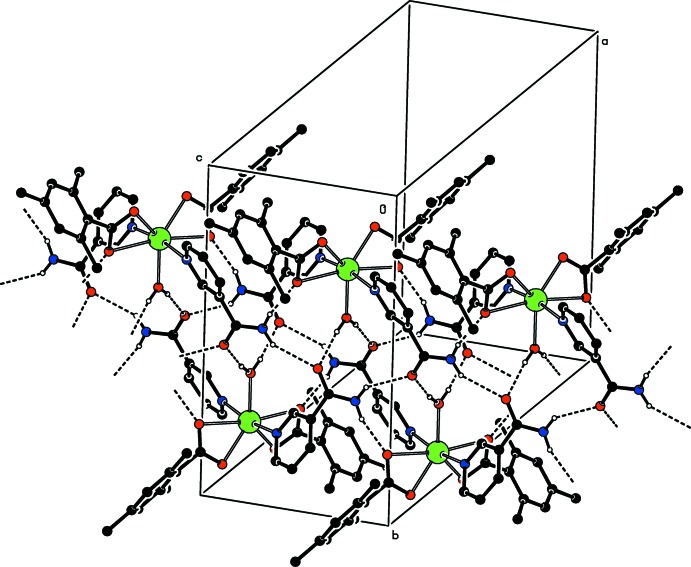
Part of the crystal structure. O—H_W_⋯O_NA_, N—H_NA_⋯O_C_ and N—H_NA_⋯O_NA_ (W = water, C = carboxyl­ate and NA = nicotinamide) hydrogen bonds, enclosing 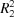
(12), 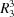
(8), 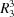
(14), 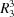
(16), 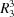
(20), 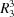
(22), 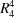
(22), 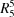
(16), 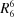
(16) and 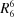
(18) ring motifs are shown as dashed lines. C-bound H atoms have been omitted for clarity.

In the crystal, the mol­ecules are linked *via* inter­molecular N—H_NA_⋯O_NA_, N—H_NA_⋯O_C_, O—H_W_⋯O_NA_ and C—H_TMB_⋯O_C_ (NA = nicotinamide, C = carboxyl­ate, W = water and TMB = 2,4,6-tri­methyl­benzoate) hydrogen bonds (Table 1 ▸) with 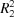
(12), 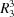
(8), 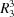
(14), 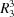
(16), 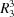
(20), 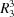
(22), 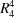
(22), 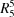
(16), 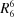
(16) and 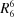
(18) ring motifs (Fig. 2 ▸), forming a three-dimensional architecture. Hydrogen-bonding and van der Waals contacts are the dominant inter­actions in the crystal packing. No significant π–π or C—H⋯π inter­actions are observed.

## Hirshfeld surface analysis   

Visulization and exploration of inter­molecular close contacts of a structure is invaluable, and this can be achieved using Hirshfeld surface (HS) analysis (Hirshfeld, 1977 ▸; Spackman & Jayatilaka, 2009 ▸). An HS analysis was carried out by using *CrystalExplorer17.5* (Turner *et al.*, 2017 ▸) to investigate the locations of atom⋯atom short contacts with the potential to form hydrogen bonds and the qu­anti­tative ratios of these inter­actions and the π-stacking inter­actions in the crystal structure of the title complex.

**Figure 3:**
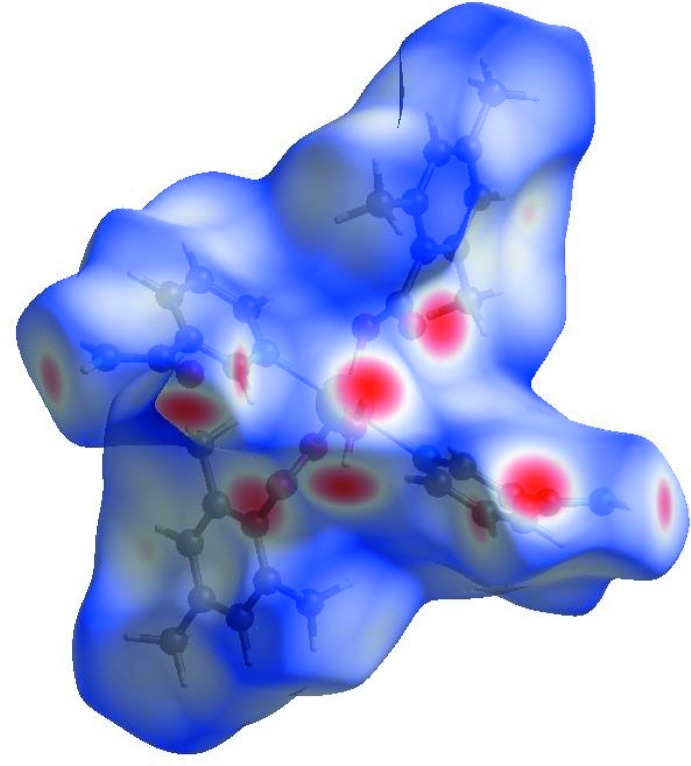
View of the three-dimensional Hirshfeld surface of the title complex plotted over *d*_norm_ in the range −0.6741 to 1.6440 a.u.

**Figure 4:**
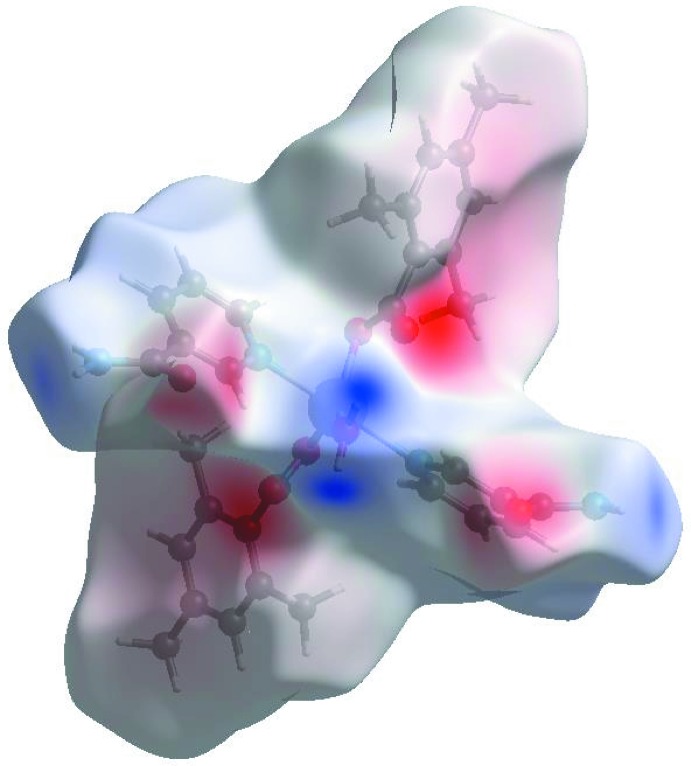
View of the three-dimensional Hirshfeld surface of the title complex plotted over electrostatic potential energy in the range −0.1379 to 0.1988 a.u. using the STO-3G basis set at the Hartree–Fock level of theory. The N—H⋯O, O—H⋯O and C—H⋯O hydrogen-bond donors and acceptors are viewed as blue and red regions around the atoms corresponding to positive and negative potentials, respectively.

**Figure 5:**
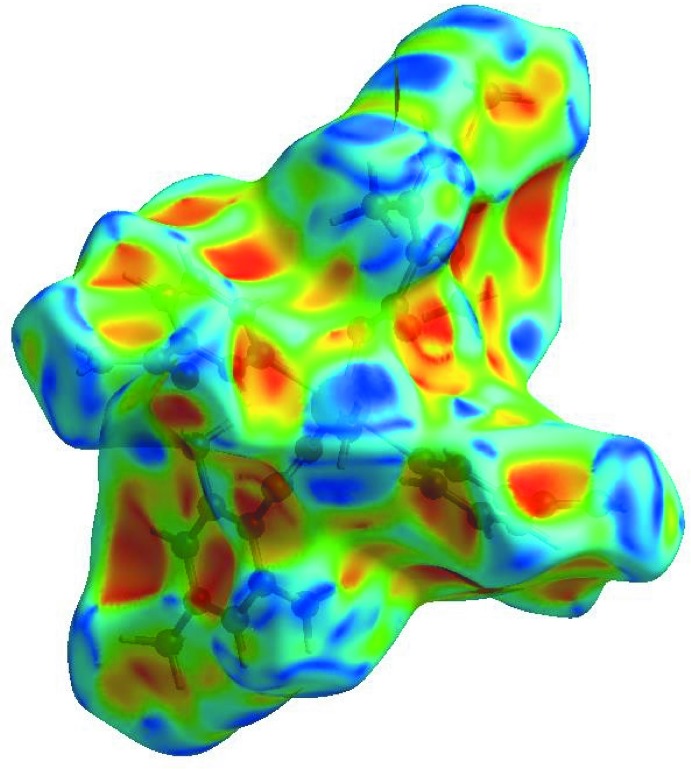
Hirshfeld surface of the title complex plotted over shape-index.

In the HS plotted over *d*_norm_ (Fig. 3 ▸), the white surface indicates contacts with distances equal to the sum of van der Waals radii, and the red and blue colours indicate distances shorter (in close contact) or longer (distinct contact) than the van der Waals radii, respectively (Venkatesan *et al.*, 2016 ▸). The bright-red spots appearing near NA-O3, TMB-O1 and O2, and hydrogen atoms H2*A*, H2*B*, H41 and H8*C* indicate their role as the respective donors and acceptors in the dominant O—H⋯O, N—H⋯O and C—H⋯O hydrogen bonds; they also appear as blue and red regions corresponding to positive and negative potentials on the HS mapped over electrostatic potential (Spackman *et al.*, 2008 ▸; Jayatilaka *et al.*, 2005 ▸) as shown in Fig. 4 ▸. The blue regions indicate the positive electrostatic potential (hydrogen-bond donors), while the red regions indicate the negative electrostatic potential (hydrogen-bond acceptors). The shape-index of the HS is a tool to visualize the π–π stacking by the presence of adjacent red and blue triangles; if there are no adjacent red and/or blue triangles, then there are no π–π inter­actions. Fig. 5 ▸ clearly suggests that there are no π–π inter­actions in (I).

**Figure 6:**
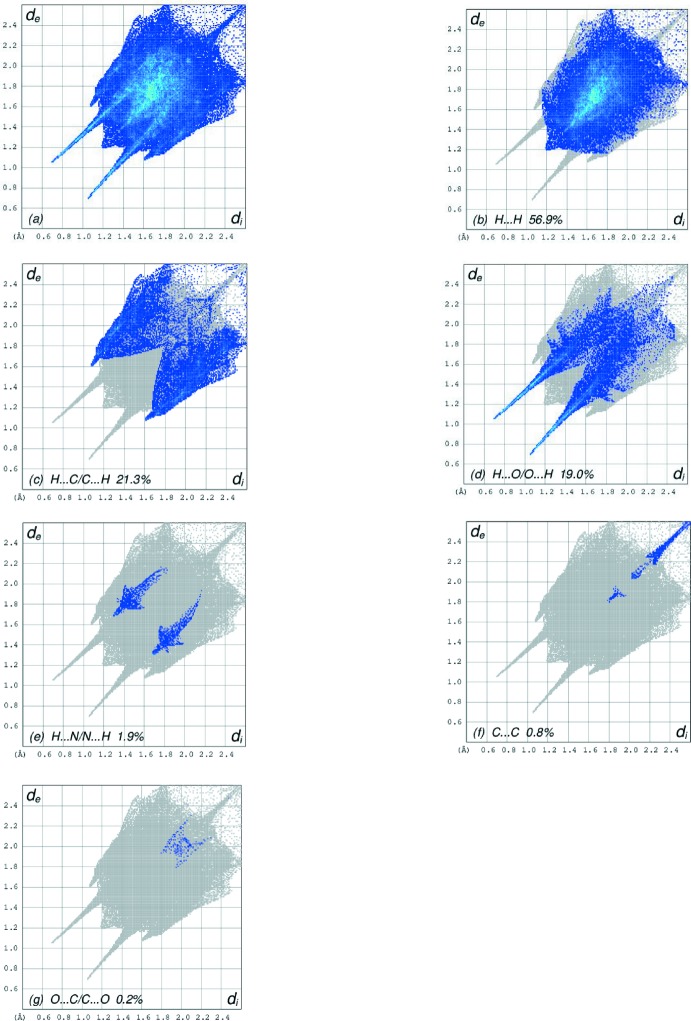
The full two-dimensional fingerprint plots for the title complex, showing (*a*) all inter­actions, and delineated into (*b*) H⋯H, (*c*) H⋯C/C⋯H, (*d*) H⋯O/O⋯H, (*e*) H⋯N/N⋯H, (*f*) C⋯C and (*g*) O⋯C/C⋯O inter­actions. The *d*_i_ and *d*_e_ values are the closest inter­nal and external distances (in Å) from given points on the Hirshfeld surface contacts.

**Figure 7:**
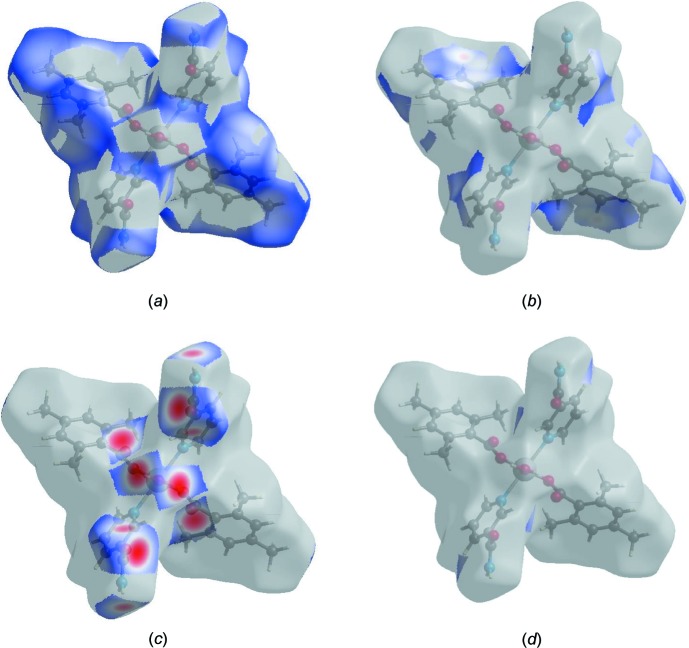
The Hirshfeld surface representations with the function *d*_norm_ plotted onto the surface for (*a*) H⋯H, (*b*) H⋯C/C⋯H, (*c*) H⋯O/O⋯H and (*d*) H⋯N/N⋯H inter­actions.

The overall two-dimensional fingerprint plot, Fig. 6 ▸ *a*, and those delineated into H⋯H, H⋯C/C⋯H, H⋯O/O⋯H, H⋯N/N⋯H, C⋯C and O⋯C/C⋯O contacts (McKinnon *et al.*, 2007 ▸) are illustrated in Fig. 6 ▸ *b*–*g*, respectively, together with their relative contributions to the Hirshfeld surface. The most important inter­action is H⋯H, contributing 56.9% to the overall crystal packing, which is reflected in Fig. 6 ▸ *b* as widely scattered points of high density due to the large hydrogen content of the mol­ecule. The single spike in the centre at *d*_e_ = *d*_i_ = 1.2 Å in Fig. 6 ▸ *b* is due to a short inter­atomic H⋯H contact (Table 2 ▸). In the absence of C—H⋯π inter­actions in the crystal, the pair of characteristic wings resulting in the fingerprint plot delineated into H⋯C/C⋯H contacts, with 21.3% contribution to the HS, Fig. 6 ▸ *c*; the pair of thin edges at *d*_e_ + *d*_i_ ∼ 1.67 Å result from short inter­atomic H⋯C/C⋯H contacts (Table 2 ▸). In the fingerprint plot delineated into H⋯O/O⋯H contacts, Fig. 6 ▸ *d*, the 19.0% contribution to the HS arises from inter­molecular O—H⋯O hydrogen bonding and is viewed as a pair of spikes with the tip at *d*_e_ + *d*_i_ ∼ 1.74 Å. The short H⋯O/O⋯H contacts are masked by strong O—H⋯O hydrogen bonding in this plot. The H⋯N/N⋯H contacts in the structure, with a 1.9% contribution to the HS, has a symmetrical distribution of points, Fig. 6 ▸ *e*, with the tips at *d*_e_ + *d*_i_ ∼ 2.96 Å arising from the short inter­atomic H⋯N/N⋯H contact listed in Table 2 ▸. The Hirshfeld surface representations with the function *d*_norm_ plotted onto the surface are shown for the H⋯H, H⋯C/C⋯H, H⋯O/O⋯H and H⋯N/N⋯H inter­actions in Fig. 7 ▸ *a*–*d*, respectively.

The Hirshfeld surface analysis confirms the importance of H-atom contacts in establishing the packing. The large number of H⋯H, H⋯C/C⋯H and H⋯O/O⋯H inter­actions suggest that van der Waals inter­actions and hydrogen bonding play the major roles in the crystal packing (Hathwar *et al.*, 2015 ▸).

## Synthesis and crystallization   

The title compound was prepared by the reaction of 3CdSO_4_·8H_2_O (0.64 g, 2.5 mmol) in water (50 ml) and nicotinamide (0.61 g, 5 mmol) in water (25 ml) with sodium 2,4,6-tri­methyl­benzoate (0.93 g, 5 mmol) in water (150 ml) at room temperature. The mixture was filtered and set aside to crystallize at ambient temperature for six weeks, giving colourless single crystals (yield: 1.42 g, 85%). Combustion analysis: found; C, 57.07, H, 5.67, N, 7.92%. Calculated: C_32_H_36_CdN_4_O_7_ C, 57.42; H, 5.43, N, 8.34%. FT–IR: 3390, 3122, 2921, 1669, 1619, 1539, 1445, 1399, 1113, 1038, 847, 731, 641 cm^−1^.

## Refinement   

Crystal data, data collection and structure refinement details are summarized in Table 3 ▸. The H atoms of the NH_2_ group and of the water mol­ecule were located in difference-Fourier maps and refined freely. The C-bound H atoms were positioned geometrically with C—H = 0.93 and 0.96 Å for aromatic and methyl H atoms, respectively, and constrained to ride on their parent atoms, with *U*_iso_(H) = *k* × *U*_eq_(C), where *k* = 1.5 for methyl H-atoms and k = 1.2 for aromatic H-atoms.

## Supplementary Material

Crystal structure: contains datablock(s) I, global. DOI: 10.1107/S2056989018001494/xu5916sup1.cif

Structure factors: contains datablock(s) I. DOI: 10.1107/S2056989018001494/xu5916Isup2.hkl

CCDC reference: 1818756

Additional supporting information:  crystallographic information; 3D view; checkCIF report

## Figures and Tables

**Table 1:** Hydrogen-bond geometry (Å, °)

*D*—H⋯*A*	*D*—H	H⋯*A*	*D*⋯*A*	*D*—H⋯*A*
N2—H2*A*⋯O3^vi^	0.89 (3)	2.26 (4)	3.047 (4)	147 (3)
N2—H2*B*⋯O2^vii^	0.81 (3)	2.03 (3)	2.830 (4)	168 (4)
O4—H41⋯O3^iii^	0.80 (3)	1.92 (3)	2.714 (3)	170 (3)
C8—H8*C*⋯O1^viii^	0.96	2.55	3.468 (5)	161

Symmetry codes: (iii) 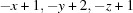; (vi) 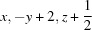; (vii) 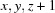; (viii) 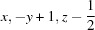.

**Table 2:** Selected interatomic distances (Å)

O1⋯H8*C* ^i^	2.55	N2⋯H13	2.75
O2⋯H2*B* ^ii^	2.03 (3)	C6⋯H14^ii^	2.80
O3⋯H41^iii^	1.92 (3)	C16⋯H41^iii^	2.85 (3)
O3⋯H2*A* ^iv^	2.26 (4)	H8*A*⋯H8*A* ^v^	2.54

Symmetry codes: (i) 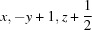; (ii) 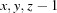; (iii) 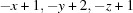; (iv) 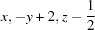; (v) 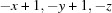.

**Table 3:** Experimental details

Crystal data	
Chemical formula	[Cd(C_10_H_11_O_2_)_2_(C_6_H_6_N_2_O)_2_(H_2_O)]
*M*_r_	701.05
Crystal system, space group	Orthorhombic, *P* *b* *c* *n*
Temperature (K)	296
*a*, *b*, *c* (Å)	23.6876 (5), 15.6711 (4), 9.0682 (2)
*V* (Å^3^)	3366.21 (13)
*Z*	4
Radiation type	Mo *K*α
μ (mm^−1^)	0.70
Crystal size (mm)	0.45 × 0.28 × 0.21

Data collection	
Diffractometer	Bruker SMART BREEZE CCD
Absorption correction	Multi-scan (*SADABS*; Bruker, 2012 ▸)
*T*_min_, *T*_max_	0.784, 0.867
No. of measured, independent and observed [*I* > 2σ(*I*)] reflections	68263, 4213, 3681
*R*_int_	0.028
(sin θ/λ)_max_ (Å^−1^)	0.669

Refinement	
*R*[*F* ^2^ > 2σ(*F* ^2^)], *wR*(*F* ^2^), *S*	0.047, 0.099, 1.32
No. of reflections	4213
No. of parameters	215
H-atom treatment	H atoms treated by a mixture of independent and constrained refinement
Δρ_max_, Δρ_min_ (e Å^−3^)	0.32, −0.47

Computer programs: *APEX2* and *SAINT* (Bruker, 2012 ▸), *SHELXS97* and *SHELXL97* (Sheldrick, 2008 ▸), *ORTEP-3 for Windows* and *WinGX* (Farrugia, 2012 ▸) and *PLATON* (Spek, 2009 ▸).

## supplementary crystallographic information

### Crystal data

**Table:**

[Cd(C_10_H_11_O_2_)_2_(C_6_H_6_N_2_O)_2_(H_2_O)]	*F*(000) = 1440
*M**_r_* = 701.05	*D*_x_ = 1.383 Mg m^−^^3^
Orthorhombic, *P**b**c**n*	Mo *K*α radiation, λ = 0.71073 Å
Hall symbol: -P 2n 2ab	Cell parameters from 9766 reflections
*a* = 23.6876 (5) Å	θ = 2.6–28.4°
*b* = 15.6711 (4) Å	µ = 0.70 mm^−^^1^
*c* = 9.0682 (2) Å	*T* = 296 K
*V* = 3366.21 (13) Å^3^	Block, colorless
*Z* = 4	0.45 × 0.28 × 0.21 mm

### Data collection

**Table:**

Bruker SMART BREEZE CCD diffractometer	4213 independent reflections
Radiation source: fine-focus sealed tube	3681 reflections with *I* > 2σ(*I*)
Graphite monochromator	*R*_int_ = 0.028
φ and ω scans	θ_max_ = 28.4°, θ_min_ = 1.6°
Absorption correction: multi-scan (*SADABS*; Bruker, 2012)	*h* = −31→31
*T*_min_ = 0.784, *T*_max_ = 0.867	*k* = −20→20
68263 measured reflections	*l* = −11→12

### Refinement

**Table:**

Refinement on *F*^2^	Primary atom site location: structure-invariant direct methods
Least-squares matrix: full	Secondary atom site location: difference Fourier map
*R*[*F*^2^ > 2σ(*F*^2^)] = 0.047	Hydrogen site location: inferred from neighbouring sites
*wR*(*F*^2^) = 0.099	H atoms treated by a mixture of independent and constrained refinement
*S* = 1.32	*w* = 1/[σ^2^(*F*_o_^2^) + (0.0171*P*)^2^ + 5.5549*P*] where *P* = (*F*_o_^2^ + 2*F*_c_^2^)/3
4213 reflections	(Δ/σ)_max_ = 0.001
215 parameters	Δρ_max_ = 0.32 e Å^−^^3^
0 restraints	Δρ_min_ = −0.47 e Å^−^^3^

### Special details

**Table:**

Geometry. All esds (except the esd in the dihedral angle between two l.s. planes) are estimated using the full covariance matrix. The cell esds are taken into account individually in the estimation of esds in distances, angles and torsion angles; correlations between esds in cell parameters are only used when they are defined by crystal symmetry. An approximate (isotropic) treatment of cell esds is used for estimating esds involving l.s. planes.
Refinement. Refinement of F^2^ against ALL reflections. The weighted R-factor wR and goodness of fit S are based on F^2^, conventional R-factors R are based on F, with F set to zero for negative F^2^. The threshold expression of F^2^ > 2sigma(F^2^) is used only for calculating R-factors(gt) etc. and is not relevant to the choice of reflections for refinement. R-factors based on F^2^ are statistically about twice as large as those based on F, and R- factors based on ALL data will be even larger.

### Fractional atomic coordinates and isotropic or equivalent isotropic displacement parameters (Å^2^)

**Table:**

	*x*	*y*	*z*	*U*_iso_*/*U*_eq_	
Cd1	0.5000	0.746100 (17)	0.2500	0.03358 (9)	
O1	0.43935 (10)	0.64030 (14)	0.1751 (3)	0.0472 (6)	
O2	0.42005 (10)	0.76163 (14)	0.0676 (3)	0.0468 (6)	
O3	0.42068 (10)	0.98712 (14)	0.6880 (3)	0.0471 (5)	
O4	0.5000	0.8932 (2)	0.2500	0.0481 (8)	
H41	0.5257 (14)	0.924 (2)	0.272 (4)	0.041 (10)*	
N1	0.44028 (11)	0.75971 (16)	0.4585 (3)	0.0392 (6)	
N2	0.41074 (13)	0.91347 (19)	0.9004 (3)	0.0434 (7)	
H2A	0.4139 (15)	0.960 (2)	0.957 (4)	0.053 (11)*	
H2B	0.4105 (15)	0.867 (2)	0.939 (4)	0.050 (11)*	
C1	0.41017 (13)	0.68404 (18)	0.0888 (3)	0.0344 (6)	
C2	0.36190 (13)	0.64076 (19)	0.0124 (3)	0.0387 (7)	
C3	0.30699 (16)	0.6685 (3)	0.0358 (5)	0.0587 (10)	
C4	0.26341 (18)	0.6243 (4)	−0.0346 (6)	0.0812 (15)	
H4	0.2264	0.6419	−0.0194	0.097*	
C5	0.2733 (2)	0.5561 (4)	−0.1254 (6)	0.0801 (15)	
C6	0.32799 (19)	0.5306 (3)	−0.1478 (5)	0.0662 (12)	
H6	0.3351	0.4847	−0.2100	0.079*	
C7	0.37317 (16)	0.5717 (2)	−0.0798 (4)	0.0475 (8)	
C8	0.43251 (18)	0.5435 (3)	−0.1127 (5)	0.0715 (13)	
H8A	0.4488	0.5186	−0.0259	0.107*	
H8B	0.4546	0.5919	−0.1425	0.107*	
H8C	0.4320	0.5021	−0.1907	0.107*	
C9	0.2941 (2)	0.7424 (4)	0.1375 (7)	0.099 (2)	
H9A	0.3084	0.7301	0.2343	0.149*	
H9B	0.2540	0.7509	0.1424	0.149*	
H9C	0.3118	0.7932	0.1004	0.149*	
C10	0.2234 (3)	0.5105 (5)	−0.1995 (8)	0.143 (3)	
H10A	0.2204	0.4535	−0.1614	0.214*	
H10B	0.2295	0.5083	−0.3041	0.214*	
H10C	0.1892	0.5411	−0.1792	0.214*	
C11	0.44160 (12)	0.82871 (18)	0.5440 (3)	0.0361 (6)	
H11	0.4655	0.8732	0.5176	0.043*	
C12	0.40932 (12)	0.83775 (17)	0.6698 (3)	0.0314 (6)	
C13	0.37296 (15)	0.7725 (2)	0.7083 (4)	0.0445 (8)	
H13	0.3509	0.7761	0.7929	0.053*	
C14	0.37015 (16)	0.7016 (2)	0.6178 (4)	0.0529 (9)	
H14	0.3455	0.6572	0.6398	0.064*	
C15	0.40397 (16)	0.6973 (2)	0.4953 (4)	0.0470 (8)	
H15	0.4017	0.6493	0.4353	0.056*	
C16	0.41406 (12)	0.91928 (18)	0.7553 (3)	0.0357 (6)	

### Atomic displacement parameters (Å^2^)

**Table:**

	*U*^11^	*U*^22^	*U*^33^	*U*^12^	*U*^13^	*U*^23^
Cd1	0.03990 (16)	0.02586 (14)	0.03497 (16)	0.000	−0.00070 (14)	0.000
O1	0.0606 (14)	0.0357 (11)	0.0452 (13)	−0.0048 (10)	−0.0166 (11)	0.0054 (10)
O2	0.0552 (14)	0.0349 (11)	0.0504 (14)	−0.0075 (10)	−0.0097 (12)	0.0037 (10)
O3	0.0675 (15)	0.0323 (11)	0.0413 (12)	−0.0071 (10)	−0.0008 (12)	0.0041 (10)
O4	0.0467 (19)	0.0269 (14)	0.071 (2)	0.000	−0.0015 (19)	0.000
N1	0.0449 (14)	0.0373 (13)	0.0356 (14)	−0.0081 (11)	0.0017 (11)	−0.0062 (11)
N2	0.0645 (19)	0.0310 (13)	0.0346 (14)	0.0058 (13)	−0.0028 (13)	−0.0019 (12)
C1	0.0408 (15)	0.0326 (14)	0.0299 (14)	−0.0032 (12)	0.0014 (12)	−0.0019 (12)
C2	0.0418 (16)	0.0375 (15)	0.0369 (16)	−0.0074 (13)	−0.0042 (13)	0.0058 (13)
C3	0.0457 (19)	0.073 (3)	0.057 (2)	−0.0045 (18)	0.0016 (17)	−0.003 (2)
C4	0.042 (2)	0.121 (4)	0.081 (3)	−0.018 (2)	−0.003 (2)	0.005 (3)
C5	0.072 (3)	0.101 (4)	0.067 (3)	−0.043 (3)	−0.016 (2)	0.001 (3)
C6	0.085 (3)	0.056 (2)	0.058 (2)	−0.030 (2)	−0.011 (2)	−0.0060 (19)
C7	0.061 (2)	0.0349 (16)	0.0465 (19)	−0.0115 (15)	−0.0078 (16)	0.0007 (14)
C8	0.074 (3)	0.057 (2)	0.083 (3)	0.010 (2)	−0.005 (2)	−0.032 (2)
C9	0.055 (3)	0.130 (5)	0.113 (5)	0.017 (3)	0.009 (3)	−0.042 (4)
C10	0.103 (4)	0.189 (7)	0.137 (6)	−0.085 (5)	−0.034 (4)	−0.022 (5)
C11	0.0391 (15)	0.0323 (14)	0.0368 (15)	−0.0100 (12)	0.0041 (13)	0.0004 (12)
C12	0.0359 (14)	0.0310 (13)	0.0274 (13)	−0.0027 (11)	−0.0015 (11)	0.0032 (11)
C13	0.0507 (19)	0.0490 (18)	0.0340 (16)	−0.0122 (15)	0.0065 (14)	0.0034 (14)
C14	0.068 (2)	0.0449 (18)	0.0457 (19)	−0.0277 (17)	0.0084 (17)	0.0038 (15)
C15	0.067 (2)	0.0339 (15)	0.0403 (17)	−0.0140 (15)	0.0006 (16)	−0.0033 (14)
C16	0.0361 (14)	0.0356 (14)	0.0353 (14)	0.0009 (11)	−0.0012 (13)	0.0013 (13)

### Geometric parameters (Å, º)

**Table:**

Cd1—O1	2.297 (2)	C4—H4	0.9300
Cd1—O1^i^	2.297 (2)	C5—C10	1.536 (6)
Cd1—O2	2.527 (2)	C6—C5	1.371 (7)
Cd1—O2^i^	2.527 (2)	C6—H6	0.9300
Cd1—O4	2.306 (3)	C7—C6	1.392 (5)
Cd1—N1	2.371 (3)	C7—C8	1.503 (5)
Cd1—N1^i^	2.371 (3)	C8—H8A	0.9600
Cd1—C1	2.759 (3)	C8—H8B	0.9600
Cd1—C1^i^	2.759 (3)	C8—H8C	0.9600
O1—C1	1.249 (4)	C11—H11	0.9300
O2—C1	1.253 (3)	C12—C11	1.380 (4)
O3—C16	1.236 (4)	C12—C13	1.382 (4)
O4—H41	0.80 (3)	C12—C16	1.499 (4)
N1—C11	1.331 (4)	C13—C14	1.382 (5)
N1—C15	1.344 (4)	C13—H13	0.9300
N2—C16	1.321 (4)	C14—H14	0.9300
N2—H2A	0.90 (4)	C15—C14	1.371 (5)
N2—H2B	0.81 (4)	C15—H15	0.9300
C1—C2	1.499 (4)	C9—H9A	0.9600
C2—C3	1.388 (5)	C9—H9B	0.9600
C2—C7	1.394 (5)	C9—H9C	0.9600
C3—C4	1.397 (6)	C10—H10A	0.9600
C3—C9	1.512 (6)	C10—H10B	0.9600
C4—C5	1.370 (7)	C10—H10C	0.9600
			
O1···H8C^ii^	2.55	N2···H13	2.75
O2···H2B^iii^	2.03 (3)	C6···H14^iii^	2.80
O3···H41^iv^	1.92 (3)	C16···H41^iv^	2.85 (3)
O3···H2A^v^	2.26 (4)	H8A···H8A^vi^	2.54
			
O1—Cd1—O1^i^	87.57 (11)	C7—C2—C1	118.9 (3)
O1—Cd1—O2	53.63 (7)	C2—C3—C4	117.9 (4)
O1^i^—Cd1—O2	137.06 (8)	C2—C3—C9	121.5 (4)
O1—Cd1—O2^i^	137.06 (8)	C4—C3—C9	120.6 (4)
O1^i^—Cd1—O2^i^	53.63 (7)	C3—C4—H4	118.8
O1—Cd1—O4	136.22 (6)	C5—C4—C3	122.3 (4)
O1^i^—Cd1—O4	136.22 (6)	C5—C4—H4	118.8
O1—Cd1—N1	85.85 (9)	C4—C5—C6	118.6 (4)
O1^i^—Cd1—N1	101.67 (9)	C4—C5—C10	119.7 (5)
O1—Cd1—N1^i^	101.67 (9)	C6—C5—C10	121.8 (5)
O1^i^—Cd1—N1^i^	85.85 (9)	C5—C6—C7	121.8 (4)
O1—Cd1—C1	26.66 (8)	C5—C6—H6	119.1
O1^i^—Cd1—C1	112.62 (9)	C7—C6—H6	119.1
O1—Cd1—C1^i^	112.62 (9)	C2—C7—C8	121.7 (3)
O1^i^—Cd1—C1^i^	26.66 (8)	C6—C7—C2	118.5 (4)
O2—Cd1—O2^i^	168.94 (10)	C6—C7—C8	119.7 (4)
O2—Cd1—C1	26.97 (7)	C7—C8—H8A	109.5
O2^i^—Cd1—C1	163.57 (8)	C7—C8—H8B	109.5
O2—Cd1—C1^i^	163.57 (8)	C7—C8—H8C	109.5
O2^i^—Cd1—C1^i^	26.97 (7)	H8A—C8—H8B	109.5
O4—Cd1—O2	84.47 (5)	H8A—C8—H8C	109.5
O4—Cd1—O2^i^	84.47 (5)	H8B—C8—H8C	109.5
O4—Cd1—N1	84.84 (6)	C3—C9—H9A	109.5
O4—Cd1—N1^i^	84.84 (6)	C3—C9—H9B	109.5
O4—Cd1—C1	110.64 (6)	C3—C9—H9C	109.5
O4—Cd1—C1^i^	110.64 (6)	H9A—C9—H9B	109.5
N1—Cd1—O2	93.81 (9)	H9A—C9—H9C	109.5
N1^i^—Cd1—O2	85.20 (9)	H9B—C9—H9C	109.5
N1—Cd1—O2^i^	85.20 (9)	C5—C10—H10A	109.5
N1^i^—Cd1—O2^i^	93.81 (9)	C5—C10—H10B	109.5
N1—Cd1—N1^i^	169.68 (12)	C5—C10—H10C	109.5
N1—Cd1—C1	89.67 (9)	H10A—C10—H10B	109.5
N1^i^—Cd1—C1	93.97 (9)	H10A—C10—H10C	109.5
N1—Cd1—C1^i^	93.97 (9)	H10B—C10—H10C	109.5
N1^i^—Cd1—C1^i^	89.67 (9)	N1—C11—C12	123.5 (3)
C1—Cd1—C1^i^	138.71 (12)	N1—C11—H11	118.3
C1—O1—Cd1	97.78 (18)	C12—C11—H11	118.3
C1—O2—Cd1	86.90 (18)	C11—C12—C13	118.6 (3)
Cd1—O4—H41	127 (2)	C11—C12—C16	118.3 (3)
C11—N1—Cd1	121.6 (2)	C13—C12—C16	123.1 (3)
C11—N1—C15	117.5 (3)	C12—C13—C14	118.3 (3)
C15—N1—Cd1	120.9 (2)	C12—C13—H13	120.8
C16—N2—H2A	121 (2)	C14—C13—H13	120.8
C16—N2—H2B	120 (3)	C13—C14—H14	120.3
H2A—N2—H2B	119 (4)	C15—C14—C13	119.5 (3)
O1—C1—Cd1	55.57 (15)	C15—C14—H14	120.3
O1—C1—O2	121.7 (3)	N1—C15—C14	122.6 (3)
O1—C1—C2	117.6 (3)	N1—C15—H15	118.7
O2—C1—Cd1	66.13 (17)	C14—C15—H15	118.7
O2—C1—C2	120.7 (3)	O3—C16—N2	124.0 (3)
C2—C1—Cd1	173.1 (2)	O3—C16—C12	119.1 (3)
C3—C2—C1	120.2 (3)	N2—C16—C12	116.9 (3)
C3—C2—C7	121.0 (3)		
			
O1^i^—Cd1—O1—C1	−160.5 (2)	N1^i^—Cd1—C1—O2	−71.26 (19)
O2—Cd1—O1—C1	−0.25 (18)	C1^i^—Cd1—C1—O1	14.27 (18)
O2^i^—Cd1—O1—C1	175.96 (17)	C1^i^—Cd1—C1—O2	−165.28 (19)
O4—Cd1—O1—C1	19.5 (2)	Cd1—O1—C1—O2	0.5 (3)
N1—Cd1—O1—C1	97.6 (2)	Cd1—O1—C1—C2	−178.5 (2)
N1^i^—Cd1—O1—C1	−75.3 (2)	Cd1—O2—C1—O1	−0.4 (3)
C1^i^—Cd1—O1—C1	−169.85 (13)	Cd1—O2—C1—C2	178.6 (3)
O1—Cd1—O2—C1	0.25 (17)	Cd1—N1—C11—C12	−177.2 (2)
O1^i^—Cd1—O2—C1	29.9 (2)	C15—N1—C11—C12	2.4 (5)
O2^i^—Cd1—O2—C1	−166.18 (18)	Cd1—N1—C15—C14	177.8 (3)
O4—Cd1—O2—C1	−166.18 (18)	C11—N1—C15—C14	−1.8 (5)
N1—Cd1—O2—C1	−81.75 (19)	O1—C1—C2—C3	118.2 (4)
N1^i^—Cd1—O2—C1	108.55 (19)	O1—C1—C2—C7	−60.7 (4)
C1^i^—Cd1—O2—C1	36.3 (5)	O2—C1—C2—C3	−60.9 (4)
O1—Cd1—N1—C11	−162.7 (3)	O2—C1—C2—C7	120.2 (3)
O1^i^—Cd1—N1—C11	110.6 (2)	C1—C2—C3—C4	−178.2 (4)
O1—Cd1—N1—C15	17.7 (3)	C1—C2—C3—C9	0.1 (6)
O1^i^—Cd1—N1—C15	−68.9 (3)	C7—C2—C3—C4	0.7 (6)
O2—Cd1—N1—C11	−109.6 (2)	C7—C2—C3—C9	179.0 (4)
O2^i^—Cd1—N1—C11	59.3 (2)	C3—C2—C7—C6	−0.3 (5)
O2—Cd1—N1—C15	70.8 (3)	C1—C2—C7—C6	178.6 (3)
O2^i^—Cd1—N1—C15	−120.2 (3)	C3—C2—C7—C8	176.8 (4)
O4—Cd1—N1—C11	−25.6 (2)	C1—C2—C7—C8	−4.3 (5)
O4—Cd1—N1—C15	154.9 (3)	C2—C3—C4—C5	−0.5 (7)
N1^i^—Cd1—N1—C11	−25.6 (2)	C9—C3—C4—C5	−178.8 (5)
N1^i^—Cd1—N1—C15	154.9 (3)	C3—C4—C5—C6	−0.2 (8)
C1—Cd1—N1—C11	−136.3 (2)	C3—C4—C5—C10	−180.0 (5)
C1^i^—Cd1—N1—C11	84.8 (3)	C7—C6—C5—C4	0.6 (7)
C1—Cd1—N1—C15	44.1 (3)	C7—C6—C5—C10	−179.6 (5)
C1^i^—Cd1—N1—C15	−94.7 (3)	C2—C7—C6—C5	−0.4 (6)
O1^i^—Cd1—C1—O1	21.1 (3)	C8—C7—C6—C5	−177.6 (4)
O1—Cd1—C1—O2	−179.6 (3)	C13—C12—C11—N1	−1.1 (5)
O1^i^—Cd1—C1—O2	−158.40 (18)	C16—C12—C11—N1	−179.1 (3)
O2—Cd1—C1—O1	179.6 (3)	C11—C12—C13—C14	−0.8 (5)
O2^i^—Cd1—C1—O1	−9.8 (4)	C16—C12—C13—C14	177.1 (3)
O2^i^—Cd1—C1—O2	170.68 (14)	C11—C12—C16—O3	36.2 (4)
O4—Cd1—C1—O1	−165.73 (18)	C11—C12—C16—N2	−143.9 (3)
O4—Cd1—C1—O2	14.72 (19)	C13—C12—C16—O3	−141.7 (3)
N1—Cd1—C1—O1	−81.4 (2)	C13—C12—C16—N2	38.2 (4)
N1^i^—Cd1—C1—O1	108.3 (2)	C12—C13—C14—C15	1.3 (6)
N1—Cd1—C1—O2	99.07 (19)	N1—C15—C14—C13	0.0 (6)

Symmetry codes: (i) −*x*+1, *y*, −*z*+1/2; (ii) *x*, −*y*+1, *z*+1/2; (iii) *x*, *y*, *z*−1; (iv) −*x*+1, −*y*+2, −*z*+1; (v) *x*, −*y*+2, *z*−1/2; (vi) −*x*+1, −*y*+1, −*z*.

### Hydrogen-bond geometry (Å, º)

**Table:**

*D*—H···*A*	*D*—H	H···*A*	*D*···*A*	*D*—H···*A*
N2—H2*A*···O3^vii^	0.89 (3)	2.26 (4)	3.047 (4)	147 (3)
N2—H2*B*···O2^viii^	0.81 (3)	2.03 (3)	2.830 (4)	168 (4)
O4—H41···O3^iv^	0.80 (3)	1.92 (3)	2.714 (3)	170 (3)
C8—H8*C*···O1^ix^	0.96	2.55	3.468 (5)	161

Symmetry codes: (iv) −*x*+1, −*y*+2, −*z*+1; (vii) *x*, −*y*+2, *z*+1/2; (viii) *x*, *y*, *z*+1; (ix) *x*, −*y*+1, *z*−1/2.
